# Supplementary material for: Pupillary Responses to Faces Are Modulated by Familiarity and Rewarding Context
Source: Brain Sci. 2021 Jun 16;11(6):794. doi: 10.3390/brainsci11060794 (PMC8235004; doi:10.3390/brainsci11060794)
Supplement: Supplementary file 1 [file brainsci-11-00794-s001.zip › brainsci-1247141-supplementary.pdf]

Supplementary Materials to:

Pupillary Responses to Faces are Modulated by Familiarity and Rewarding Context

Magdalena Matyjek [1,2,*], Mareike Bayer [1,2] and Isabel Dziobek [1,2]

Table S1. Pairwise comparisons for the effect of face type on the subject ratings of reward value in both tasks (with Holm correction).

Contrast	Est.	Std. Error	z value	p value
celebrity.Game - stranger.Game	10.114	4.298	2.353	0.129
celebrity.Passive_task - celebrity.Game	-12.014	3.959	-3.035	0.019
celebrity.Passive_task - stranger.Passive_task	-26.886	4.298	-6.255	0.000
experimenter.Game - celebrity.Game	20.714	4.771	4.342	0.000
experimenter.Game - stranger.Game	30.829	4.112	7.497	0.000
experimenter.Passive_task - celebrity.Passive_task	-3.186	4.771	-0.668	0.979
experimenter.Passive_task - experimenter.Game	-35.914	3.959	-9.072	0.000
experimenter.Passive_task - stranger.Passive_task	-30.071	4.112	-7.313	0.000
stranger.Passive_task - stranger.Game	24.986	3.959	6.312	0.000

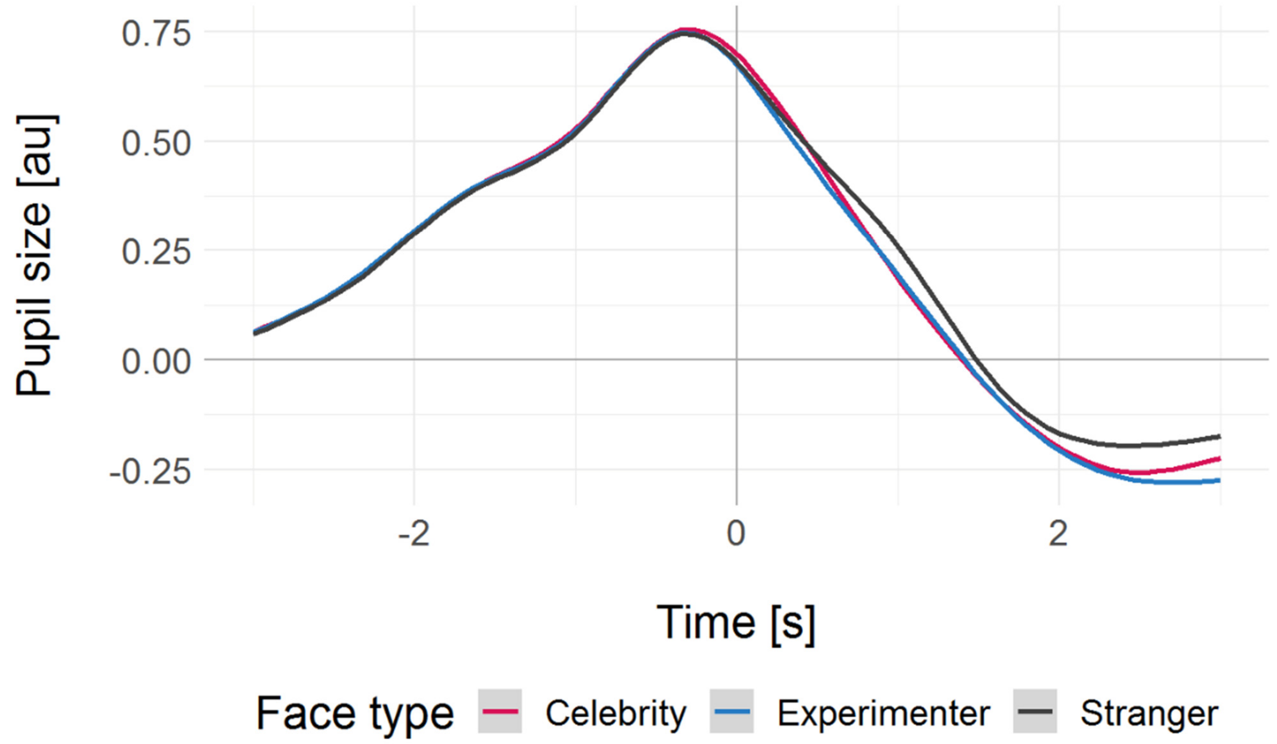

Figure S1. Plot of the pupillary responses across face types in the game - time-locked to the onset of the face presentation.

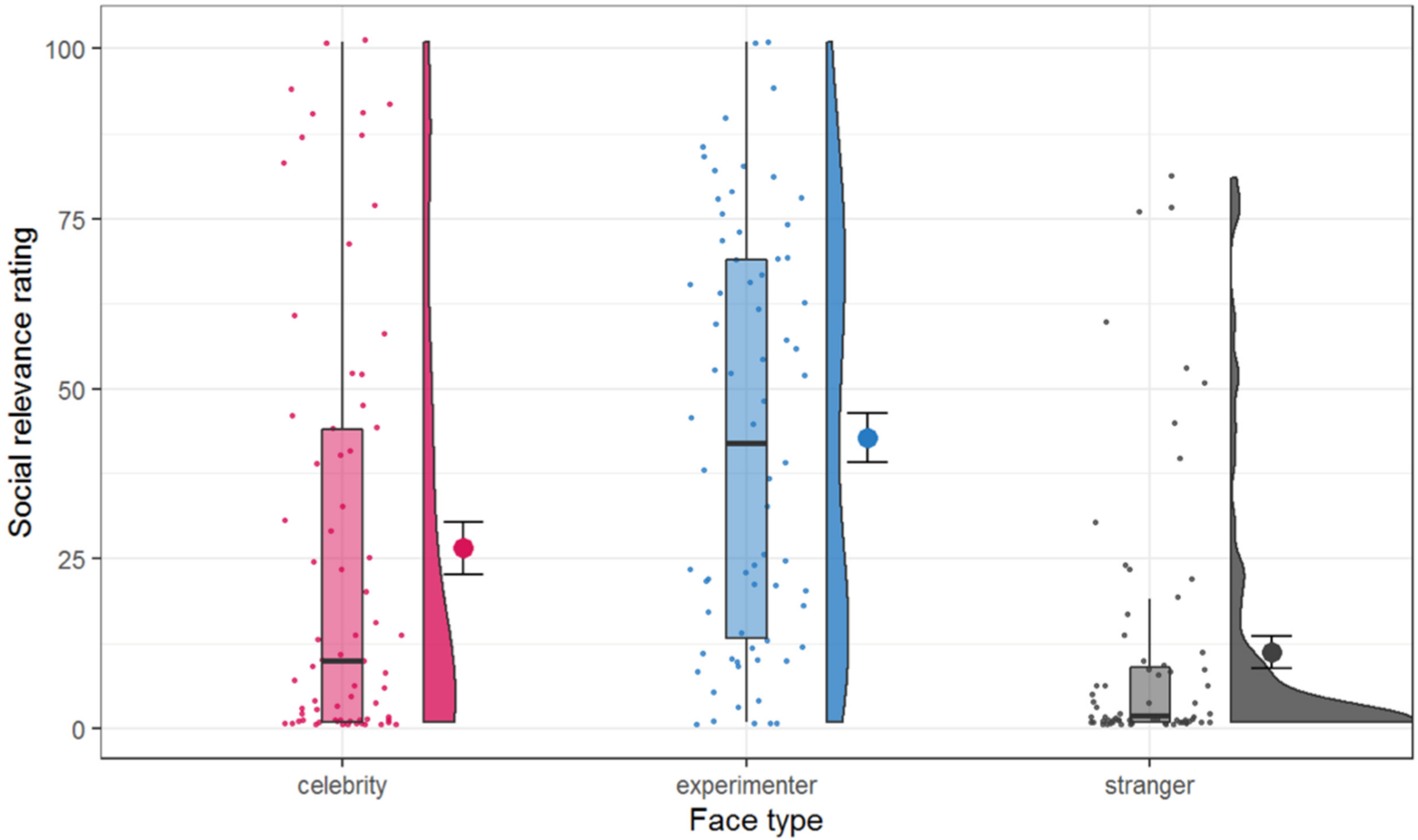

Figure S2. Subjective ratings of feedback's social relevance in the game and the passive task. The plot portraits: raw data points, boxplot, violin plot, and means with error bars marking standard deviation.

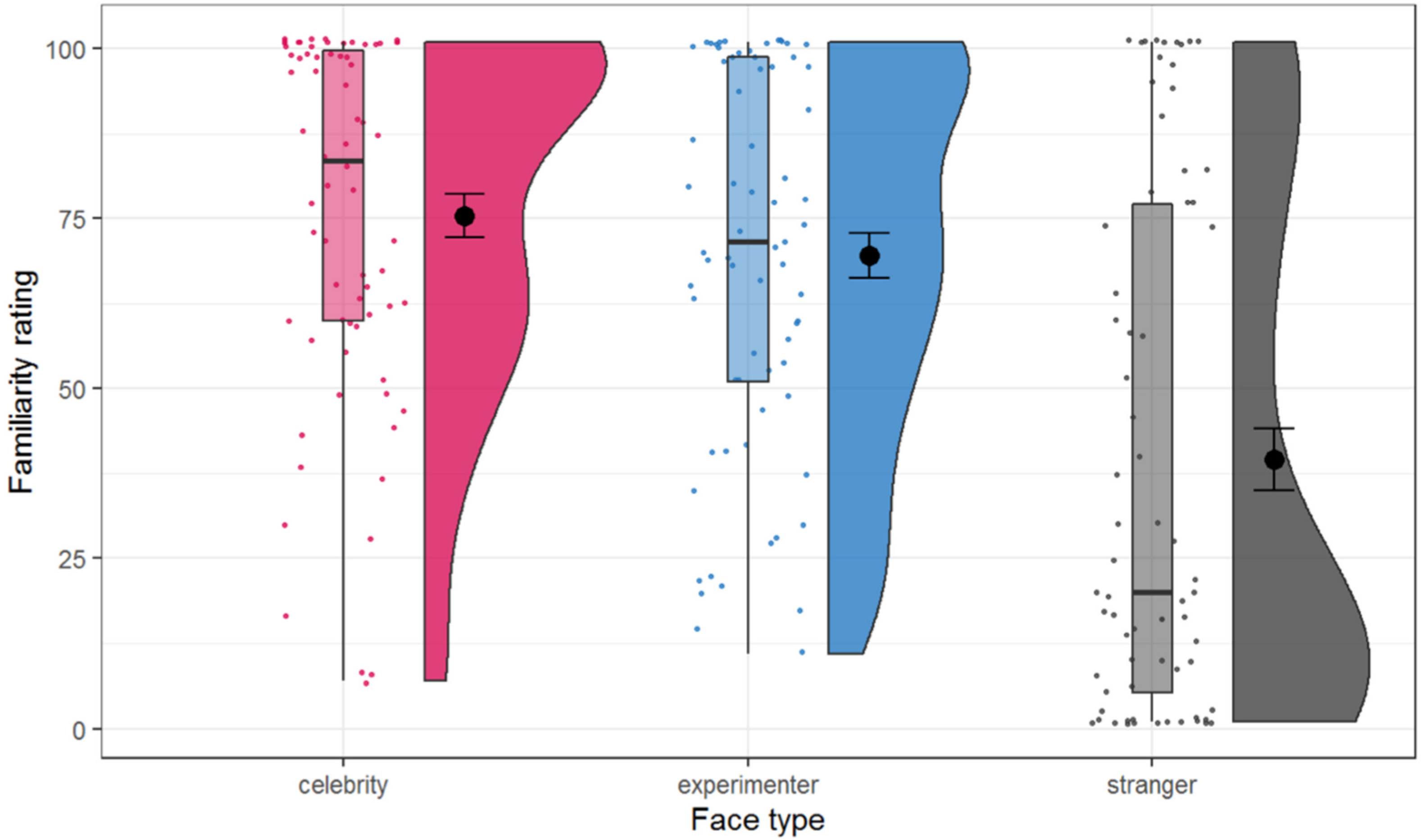

Figure S3. Subjective ratings of feedback's familiarity in the game and the passive task. The plot portraits: raw data points, boxplot, violin plot, and means with error bars marking standard deviation.
